# Quality of life, perceived stress, and use of school-based stress management interventions in high school students: a mixed-methods study during and after COVID-19

**DOI:** 10.3389/fpubh.2025.1658346

**Published:** 2025-12-11

**Authors:** Dennis John, Sebastian Otttmann, Karl-Hermann Rechberg, Teresa O‘Rourke, Rüdiger Pryss, Thomas Probst

**Affiliations:** 1 Institute for Applied Research and Evaluation, Lutheran University of Applied Sciences, Nuremberg, Germany; 2 Department for Psychosomatic Medicine and Psychotherapy, University for Continuing Education Krems, Krems an der Donau, Austria; 3 Institute of Clinical Epidemiology and Biometry, University of Würzburg, Würzburg, Germany; 4 Division of Psychotherapy, Department of Psychology, University of Salzburg, Salzburg, Austria

**Keywords:** perceived stress scale, stress levels, quality of life, COVID-19 pandemic, mixed-methods, stress management interventions, school-based mental health promotion

## Abstract

**Introduction:**

Key objectives of this study include understanding time trends in stress, understanding the associations of quality of life (QoL) and stress levels, and determining how these factors influence high school students’ engagement with school-based stress management programs.

**Methods:**

To address these aims, two complementary studies were conducted. Study 1 included two survey waves (2020 and 2021) to examine stress and QoL among German high school students. Stress levels and QoL across psychological, social, familial, and academic domains were assessed using the Perceived Stress Scale (PSS-4) and the KIDSCREEN questionnaire. Study 2 used a mixed-methods approach (paper-based questionnaire and focus groups) to explore the relationship between PSS-4 scores, school-related QoL indicators, and voluntary participation in a school-based stress management intervention day conducted in 2023.

**Results:**

Results show that stress levels among high school students increased during the COVID-19 pandemic and that low QoL in the school domain predicted higher stress. Moreover, low school-related well-being was associated with more frequent use of school-based stress management interventions.

**Discussion:**

These findings suggest that demand-tailored stress management programs in high schools could help students better cope with high stress levels—particularly those experiencing low QoL in the school setting.

## 1 Introduction

### 1.1 Stress

Selye introduced the concept of stress as the “nonspecific response of the body to any demand made upon it” (1). According to the transactional model of stress proposed by (2), two cognitive processes - primary and secondary appraisal - determine an individual’s stress experience. Primary appraisal involves the individual’s assessment of the situation’s relevance and potential threats, while secondary appraisal concerns the evaluation of the individual’s perceived ability to manage the situation. Stress arises when the situation is considered relevant, and the individual perceives the own resources as inadequate to cope with it (3). A commonly used stress questionnaire, also employed in this study, is the Perceived Stress Scale (PSS (4)). The PSS measures subjective stress perceptions concerning a specific time period, in accordance with Lazarus’ stress model.

The COVID-19 pandemic and associated containment measures, such as lockdowns and social distancing, created uncertainty and substantial disruptions to daily life globally (5). High-school students faced home-schooling aimed at reducing virus transmission, necessitating abrupt adjustments to daily routines and often resulting in a loss of social contact. Consequently, this led to elevated stress levels among both students and their parents (6). The negative impacts of the pandemic on well-being and mental health among high-school students have become increasingly evident (7). In Germany, Israel, Poland, and Slovenia indicators of mental health problems such as symptoms of depression and anxiety significantly increased after the start of the COVID-19 pandemic and have stayed at a high level since (8). Another study using a representative adolescent and parent sample found significantly higher stress levels measured by PSS 1 month into the pandemic compared to pre-pandemic levels (9). Elevated stress among adolescents was linked to various stressors across life domains, such as financial concerns, increased parental stress, procrastination, limited access to emotion regulation strategies, and prolonged home confinement during lockdowns (10). In February 2021, 1 month after the start of home-schooling measures in Austria, about half of the surveyed students reported a moderate level of stress measured with PSS, and one-third reported a high level of stress (11). By June 2021, several months later, significant reductions in stress were observed among both girls and boys, though levels remained above pre-pandemic benchmarks (12). These improvements in subjective stress perception could relate to the reopening of schools, approaching summer holidays, relaxation of public health measures, and declining COVID-19 infection rates.

### 1.2 Quality of life in different life domains

Stress negatively impacts quality of life (QoL). QoL is understood as comprising distinct yet interrelated life domains, shaped by an individual’s perceptions, interpretations, beliefs, and experiences (13). A commonly used questionnaire to assess QoL in children and adolescents, also employed in this study, is the KIDSCREEN questionnaire (14). QoL is therefore viewed as a multidimensional construct whose evaluation relies primarily on a person’s subjective appraisal of well-being and functional status across these domains. In public-health research, QoL has become a key indicator for gaging health-related burdens, stressors, and concerns within specific life domains among adolescents (15). During the COVID-19 pandemic, various QoL domains such as psychological well-being (e.g., mood, self-esteem), social relationships (e.g., peer interactions), family environment (e.g., parent–child conflict), and school life (e.g., academic achievement) were adversely impacted, contributing to elevated stress in these areas (16). According to the transactional model of stress (2), students’ appraisal of their own ability to cope with stressors may have been reduced during the pandemic due to decreased social support, disrupted daily routines, increased uncertainty, and situations perceived as more consequential for their future (3, 17). A representative survey of Austrian students conducted in February 2021 identified school-related stressors as the principal burden, followed by diminished psychological well-being and restrictions on social relationships; only about 5% of respondents reported no future concerns (18). A qualitative study conducted in spring of 2021, 1 year after the onset of the pandemic, examined stress-related changes in QoL in different life domain among Austrian students aged 14–20 years (10). The most frequently reported stressors involved school-related issues such as feeling overwhelmed, performance pressure, lack of progress, and fear of failure which were appraised as more relevant during that time due to future concerns such as reduced job security; additional stressors included personal and interpersonal concerns. In Switzerland, students most commonly reported QoL declines during the first lockdown stemming from (1) the inability to spend time with friends or family in person, (2) the suspension of social activities and normal routines, and (3) the postponement or cancelation of important plans (19). Taken together, evidence of pandemic-related declines in QoL across psychological, social, family, and school domains underscores the urgent need for targeted support strategies. One promising approach to mitigating these stress-related impacts is the implementation of school-based stress management interventions.

### 1.3 School-based stress management interventions

As declines in QoL is an indicator for reduced coping resources and heightened vulnerability to stress, targeted interventions delivered in the school context can play a crucial role in enhancing students’ resilience and promoting adaptive stress appraisals (2). School-based stress management interventions include different methods such as relaxation exercises, cognitive behavioral techniques, time management skills or social support strategies (20). Programs range from short-term activities, such as a single “stress management action day,” to long-term initiatives embedded within the curriculum (21). Reviews and meta-analyses report small- to medium-sized effects of these interventions on students’ capacity to handle everyday stressors (22, 23). To prevent further increase in stress levels after COVID-19 several initiatives have called for more evidence-based intervention programs in schools with a particular focus on mental health (24). During 2021, 47% of surveyed students expressed a desire for professional mental health support, and 16.8% wished to gain a better understanding of mental health issues and feelings of overwhelm at school, which corresponds with the motivation to increase appraisals such as feeling able to cope with situations (25). Among German university students, lower perceived stress correlated with more frequent preventive health behaviors during the pandemic (26). In practice, many schools have (re-)initiated stress management programs to mitigate the pandemic’s adverse effects (27). Nevertheless, a clearer understanding of how stressors across different life domains influence adolescents’ acceptance and use of such interventions is essential for demand-oriented planning. Stressors linked to psychological well-being, social relationships, family dynamics, and academic achievement may differentially affect students’ willingness to participate voluntarily in school-based stress management activities. Insight into these relationships will allow educators and policymakers to refine the content and format of interventions, ensuring they are tailored effectively to the needs of specific student target groups in the post-pandemic context.

### 1.4 Objective

While acute stress is an adaptive response to unpredictable changes in the environment, chronic stress can have detrimental effects on several outcomes in high school students (28). Therefore, the first aim of this study was to investigate the longitudinal change of perceived stress during the COVID-19 pandemic in high school students in Germany. Taking the vulnerability-stress model of psychopathology (29) into consideration, it is likely that students with low QoL may show more severe increase in stress levels due to the COVID-19 pandemic. Thus, a further aim of the study was to identify domain specific QoL indicators for high levels of stress among high school students. Finally, the use of school-based stress management interventions differs between target groups among high school students (22). Accordingly, a third aim of the study was to investigate which life domain specific QoL indicators affect the use of stress management interventions within schools and which factors facilitate this use.

To summarize, the following research questions (RQ) are addressed:

*RQ1*: How did the perceived stress level in high school students change between the years 2021 and 2022 during the COVID-19 pandemic?

*RQ2*: Which life domain specific QoL indicators predict stress levels in high school students during and after the COVID-19 pandemic?

*RQ3*: Which school-related QoL indicators are related to stress levels in high school students after the COVID-19 pandemic?

*RQ4*: Which school-related QoL indicators are associated with high school students’ use of school-based stress management interventions?

*RQ5*: Which factors facilitate the use of school-based stress management interventions?

## 2 Methods

### 2.1 Study 1

#### 2.1.1 Study design and participants

Study 1 included two measurement points (Wave 1 in 2020 and Wave 2 in 2021). Students could allow their data from the two waves to be matched by generating an individual code. Because not all students provided the necessary information to enable matching, we report (a) cross-sectional results for each wave and (b) longitudinal results for the subset of students whose responses could be matched. Data were collected at school, and online questionnaires were administered at both time points. The school administration supplied teachers with the survey link and detailed instructions. The teachers distributed the link to their students. Students completed the questionnaire during class using their own devices. All items were non-mandatory, so students were free to skip any question.

Of the 439 students enrolled at a German high school (Gymnasium), 345 students in grades 5 through 12 participated in the first wave of data collection (July 2020). In the second wave (June 2021), 271 students from the same school (grades 5–12) took part. Seventy-seven students provided the information needed to match their responses across both waves, allowing for longitudinal analyses. Parents were informed about the study in advance via a letter, and students gave informed consent before completing the questionnaires. The sample description of waves 1 and 2 are provided in Tables 1, 2 for the cross-sectional and the longitudinal sample.

**Table 1:** Sample description study 1.

Variable	Wave 1 (*N* = 345) *M* (SD) [SE]	Wave 2 (*N* = 271) *M* (SD) [SE]	Wave 1, longitudinal subsample (*N* = 77) *M* (SD) [SE]	Wave 2, longitudinal subsample (*N* = 77) *M* (SD) [SE]
Perceived stress scale (PSS-4)	1.40 (0.76) [0.04]	1.70 (0.82) [0.06]	1.17 (0.70) [0.08]	1.60 (0.82) [0.10]
QoL autonomy and parent relation	4.36 (0.60) [0.03]	–	4.47 (0.50) [0.06]	–
QoL social support and peers	4.04 (0.64) [0.04]	–	4.07 (0.70) [0.08]	–
QoL school environment	3.73 (0.75) [0.04]	–	3.99 (0.67) [0.08]	–
QoL psychological well-being	3.36 (0.72) [0.04]	–	3.44 (0.66) [0.08]	–
Grade level	7.56 (1.94) [0.11]	8.73 (1.38) [0.08]	7.45 (1.74) [0.20]	8.43 (1.69) [0.20]

M, mean; SD, standard deviation; SE, standard error of the mean. A dash (−) indicates that the variable was not collected in that wave.

**Table 2:** Gender distribution across samples in study 1.

Variable	Wave 1 (2020, *N* = 345)	Wave 2 (2021, *N* = 271)	Longitudinal subsample (*N* = 77)
Male	200 (58.0%)	146 (67.3%)	58 (75.3%)
Female	141 (40.9%)	70 (32.3%)	19 (24.7%)
Diverse	4 (1.2%)	1 (0.4%)	0 (0%)

Percentages may not total 100% because of rounding.

#### 2.1.2 Measures

*Perceived stress scale*: The 4-item short form of the Perceived Stress Scale [PSS-4 (4)], was used in this study to operationalize stress levels. The four PSS-Items were scored on a Likert scale ranging from 0 to 4. Higher sum scores indicate higher stress levels. The PSS is a validated measure for stress levels in adolescent populations older than 12 years (30, 31). The PSS was administered at both waves. Descriptive statistics are presented in Table 1. Cronbach’s alpha was *α* = 0.60 in wave 1 and *α* = 0.75 in wave 2. McDonald’s *ω* and corrected item–total correlations are presented in Appendix 1. *Domain specific quality of life (QoL)*: QoL in four life domains was measured with a short form of the KIDSCREEN questionnaire at wave 1 only (14). The following subscales were included: 4 items on psychological well-being (e.g., In the last week I have been happy with the way I am; Cronbach’s alpha was *α* = 0.73), 4 items on social support and peers (e.g., In the last week I had fun with my friends; Cronbach’s alpha was *α* = 0.60), 4 items on autonomy and parent relation (e.g., In the last week I got along with my parents; Cronbach’s alpha was *α* = 0.72) and 4 items on school environment (i.e., In the last week I got on well at school; Cronbach’s alpha was *α* = 0.56). The 5-point Likert-Scale ranged from never to always. Higher scores indicate higher QoL. The KIDSCREEN questionnaire has robust psychometric properties, with high discriminatory power and intern consistency of subscales (32). Descriptive statistics are presented in Table 1.

#### 2.1.3 Data analysis

IBM SPSS and R were used for statistical analyses. To address the RQ1 a *t*-test for dependent samples was performed. To address RQ2 a hierarchical linear regression model was used, with predictor variables and control variables entered block-wise. All statistical tests were 2-tailed, and the significance level was set to *p* < 0.05. For RQ1 and RQ2 the dependent variable was PSS. For RQ2, the predictor variables were QoL domains (psychological well-being, social support and peers, autonomy and parent relation, and school environment) as well as PSS in the year 2020 and the control variables were gender and grade level. We assessed multicollinearity by computing variance inflation factors (VIFs) for all predictors (reported in Appendix 2). Residual diagnostics included visual inspection of standardized residual Q–Q plots (normality) and residuals-versus-fitted plots (homoscedasticity), shown in Appendix 2. VIFs did not indicate problematic multicollinearity, and residual diagnostics did not reveal substantive deviations from normality or homoscedasticity.

Per-variable missingness is reported in Appendix 3. Results of Little’s test for Missing Completely at Random (MCAR) are reported in Appendix 3. Given the low levels of missing data and the non-significant Little’s test result consistent with MCAR we used listwise deletion in the regression analyses. Under MCAR and with small amounts of missingness, complete-case analysis yields unbiased estimates.

### 2.2 Study 2

#### 2.2.1 Study design and participants

In October 2023, the high school organized a school-wide stress management day for students in grades 7–12 to strengthen their stress resilience. During the event, students could voluntarily take part in up to three stress management activities. The program offered sessions on different stress management activities such as yoga, qigong, mindfulness and awareness, progressive muscle relaxation, and strategies for coping with exam anxiety (for further information see Appendix 4). All course instructors hold a professional qualification in the psychosocial field as well as additional training in a stress management method. The selection of instructors was carried out in accordance with the German Prevention Act and based on the criteria outlined in the Prevention Guideline of the German Association of Statutory Health Insurance Funds. All course instructors were external and not teachers at the school.

The study employed two measurement points: one to collect quantitative data and another to gather qualitative data.

Quantitative part: Data for the quantitative part were collected at school with a paper-and-pencil questionnaire. Students completed the survey during class time, either on the action day itself or within the following few days. Of the 307 students enrolled from grades 7–12, 281 submitted a completed questionnaire during that period. A detailed description of the sample (gender and grade level) is shown in Tables 3, 4. Parents received an information letter beforehand, and students provided informed consent before participating. Since the questionnaire was paper-and-pencil based, students could skip any item.

**Table 3:** Sample description study 2.

Variable	2023 stress management action day (*N* = 281)
Perceived stress scale (PSS-4)	1.64 (0.73) [0.05]
School-related QoL well-being	3.03 (1.24) [0.08]
School-related QoL school performance	2.68 (1.24) [0.07]
School-related QoL parental support	3.80 (1.32) [0.08]
School-related QoL peers	4.41 (0.90) [0.05]
Grade level	8.73 (1.38) [0.08]

M, mean; SD, standard deviation; SE, standard error of the mean. Descriptive statistics for the stress-management action-day sample (2023).

**Table 4:** Gender distribution in study 2.

Gender	*N* (%)
Male	110 (39.7%)
Female	152 (54.9%)
Diverse	15 (5.4%)

Percentages may not total 100% because of rounding.

Qualitative part: Eight to 10 days after the stress management day, six focus groups were conducted with students in grades 7, 9, and 11 (two groups per grade). Each group included a maximum of 12 students. The school administration selected three classes and each class was subsequently divided into two subgroups. Participation was strictly voluntary and students decided which subgroup they opted for. Discussions were held during two consecutive 45-min sessions in a dedicated learning room; only the students and the moderator were present. All focus groups were held by the same moderator (KR, third author of this article), who had prior training and experience in conducting qualitative focus groups. To guide the conversation, the moderator used prompt cards on which students first wrote individual responses to specific questions. These cards were then pinned to a board, and the students were invited to comment on their own and others’ answers. All sessions were audio-recorded and transcribed verbatim. The focus groups were conducted in German, and the quotations cited in this article were translated into English by the first author.

#### 2.2.2 Measures

##### 2.2.2.1 Quantitative part

The questionnaire included the following scales and items:

###### 2.2.2.1.1 Perceived stress scale

The short form of the Perceived Stress Scale PSS-4 was used as in study 1. Cronbach’s alpha was *α* = 0.61 in the current sample. Descriptive statistics are presented in Table 3. McDonald’s *ω* and corrected item–total correlations are presented in Appendix 1.

###### 2.2.2.1.2 School-related quality of life

QoL in the school domain was measured using the question, ‘What causes you stress at school…?’ for each of the following dimensions: one item on school-related well-being (‘my own expectations of myself’), one item on school-related social support and peers (‘conflicts with other classmates’), one item on school-related parental support (‘that my parents are not satisfied with my grades’), and one item on school performance (‘mastering the learning material’). The 5-point Likert scale ranged from ‘very often’ to ‘never’. Higher scores indicate higher QoL in the respective school-related domain. Descriptive statistics are presented in Table 3.

###### 2.2.2.1.3 Use of stress management interventions

The number of stress management interventions that a student took part in during the action day was counted in a variable ranging from 0 (student participated in no activity) to 3 (student participated in 3 activities). The mean number of interventions students took part in was 1.53 (SD = 1.13). Higher numbers indicate that students took part in more stress management interventions during the stress management action day.

##### 2.2.2.2 Qualitative part

The questions in the focus groups were as follows:

- How did you as a learner experience the action day on stress management?
- What have you learned during the action day?
- Have you already applied something you learned during the stress management day in your daily life?

These responses were then collected and reflected upon by the group.

#### 2.2.3 Data analysis

##### 2.2.3.1 Quantitative data

IBM SPSS and R were used for statistical analyses. To address the RQ 3, hierarchical linear regression models were used, with predictor variables (school-related QoL well-being, school-performance, parental support, and peers) and control variables (gender, grade-level) entered block-wise. To address the RQ 4, hierarchical linear regression models were used, with predictor variables (school-related QoL well-being, school-performance, parental support, and peers) and control variables (gender, grade-level) entered block-wise. All statistical tests were 2-tailed, and the significance level was set to *p* < 0.05. For RQ3, the dependent variable was PSS. For RQ4, the number of attended stress management interventions was the dependent variable. We assessed multicollinearity by computing variance inflation factors (VIFs) for all predictors (reported in Appendix 2). Residual diagnostics included visual inspection of standardized residual Q–Q plots (normality) and residuals-versus-fitted plots (homoscedasticity), shown in Appendix 2. VIFs did not indicate problematic multicollinearity, and residual diagnostics did not reveal substantive deviations from normality or homoscedasticity.

Per-variable missingness is reported in Appendix 3. Results of Little’s test for Missing Completely at Random (MCAR) are reported in Appendix 3. Given the low levels of missing data and the non-significant Little’s test result consistent with MCAR we used listwise deletion in the regression analyses. Unbiased estimates can be expected for complete-case analysis under MCAR and with small amounts of missingness.

##### 2.2.3.2 Qualitative data

The focus groups were analyzed following established approaches for qualitative content analysis (33, 34).

First, the text passages from the focus group transcripts that referred to the research questions, i.e., the coding units, were deductively selected to address the research questions related to the stress management day: What are the students’ overall impressions of the stress management day? What experiences do students report in relation to the stress management interventions? Which aspects are addressed in the students’ subsequent reflections on the interventions?

In a second step, the coding units were coded, and categories were inductively derived. Two main categories emerged: (1) students’ overall impressions of the stress management day and (2) factors that facilitate the implementation of stress management methods in students’ daily lives.

In a third step, the coding units were subsequently organized into the following subcategories: (1a) relaxing, (1b) changed their own perspective on stressful events, (1c) changed perspective of teachers on students’ stress experiences, (1d) change from the regular school routine and (1e) stress management activities within the school setting, to which students had had little exposure up to now, which belonged to the main category students’ overall impressions of the stress management day, and (2a) having fun, (2b) easy and quick to learn methods, (2c) transferable into everyday life, and (2d) clear and understandable objectives, which represented subcategories of the main category factors that facilitate the implementation of stress management methods in students’ daily lives.

In a fourth step, a coding manual was developed by the first coder (KR, third author). The first coder trained the second coder (DJ, first author). Subsequently, both coders independently assigned the coding units to the subcategories. Intercoder reliability was calculated (*κ* = 0.857; *p* < 0.001), with the κ value classified as ‘almost perfect’ (35). Codes on which the two coders did not agree were subsequently discussed and, by consensus, assigned to one of the subcategories. In accordance with these discussions, the coding manual was revised.

### 2.3 Integration of quantitative and qualitative components

In Study 2, we applied a complementary mixed-methods approach in which quantitative and qualitative components addressed distinct but related research questions (36). The quantitative analyses (RQ4) focused on identifying associations between school-related QoL indicators and students’ use of school-based stress management interventions. In contrast, the qualitative analyses (RQ5) explored students’ subjective perspectives to understand which factors they perceived as facilitating the use of such interventions. Although the two strands were not directly integrated into a single analysis, their complementary findings were considered jointly at the interpretation stage to provide a more comprehensive understanding of students’ engagement with school-based stress management programs.

## 3 Results

### 3.1 Study 1

*RQ1*: How did perceived stress levels in high school students change between the years 2021 and 2022 during the COVID-19 pandemic?

**Figure 1:**
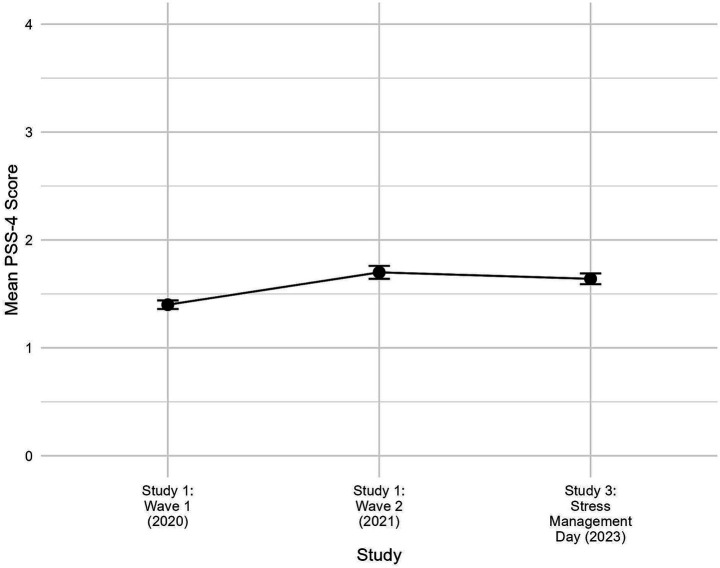
Line graph showing mean scores of perceived stress scale (PSS-4) across study 1 and 2. All available data for each wave were used; Study 1: Wave 1 (*N* = 345), Wave 2 (*N* = 271); Study 2: Stress management action day (*N* = 281). Error bars represent ± 1 Standard error.

Mean values for PSS increased from the year 2020 (*M* = 1.40, SD = 0.76) to the year 2021 (*M* = 1.68, SD = 0.82) for the cross-sectional sample. The results of the *t*-test (t df) = −3.65 (67), *p* < 0.01, Cohen’s D = −0.53 for the longitudinal sample showed significantly higher PSS values in the year 2021 (*M* = 1.60, SD = 0.82) than in the year 2020 (*M* = 1.17, SD = 0.70) indicating an increase in stress levels during the COVID-19 pandemic years 2020 and 2021 in high school students. Mean PSS scores are depicted in Figure 1.

*RQ2*: which life domain specific QoL indicators predict stress levels in high school students during and after the COVID-19 pandemic?

Table 5 shows the results of a cross-sectional linear regression model examining the associations between life domain-specific QoL (KIDSCREEN domains *psychological well-being*, *social support and peers*, *autonomy and parent relation* as well as *school environment*) and stress levels in 2020. All standardized regression weights of the domain-specific QoL were significantly associated with stress levels in 2020 showing that high levels in QoL were linked to lower stress levels. QoL in the school environment showed the strongest negative association with stress levels. Effects remained stable after controlling for gender and grade level.

**Table 5:** Cross-sectional linear regression predicting perceived stress (PSS-4) from quality of Life (QoL) indicators, Wave 1 (2020).

Parameter	Model 1†			Model 2‡		
	Std. *β*	95% CI	*p*	Std. *β*	95% CI	*p*
Intercept	−0.00	−0.08 – 0.08	< 0.001	0.05	−0.06 – 0.15	< 0.001
QoL autonomy and parent relation	−0.14	−0.22 – −0.05	< 0.001	−0.14	−0.22 – −0.05	< 0.001
QoL social support and peers	−0.13	−0.22 – −0.04	< 0.001	−0.12	−0.21 – −0.04	0.010
QoL school environment	−0.46	−0.55 – −0.36	< 0.001	−0.49	−0.58 – −0.39	< 0.001
QoL psychological well-being	−0.23	−0.32 – −0.13	< 0.001	−0.22	−0.31 – −0.13	< 0.001
Grade level	–	–	–	−0.06	−0.15 – 0.02	0.130
Gender (ref. = Male)	–	–	–			
Female	–	–	–	−0.10	−0.27 – 0.06	0.230
Diverse	–	–	–	−0.59	−1.42 – 0.23	0.160
Observations	297			297		
*R*^2^/adj. *R*^2^	0.52/0.51			0.52/0.51		

†Model 1 includes only QoL predictors. ‡Model 2 additionally adjusts for grade level and gender. Std. β = standardized beta coefficient; CI = confidence interval; ref. = reference category. Observations after list-wise deletion of missing values.

Table 6 shows the results of a longitudinal linear regression model with life domain-specific QoL (KIDSCREEN domains *psychological well-being*, *social support and peers*, *autonomy and parent relation* as well as *school environment*) and stress levels in 2020 were associated with stress levels in the year 2021 the results indicate that QoL in the school environment assessed in 2020 was a significant predictor of stress levels in 2021, with higher QoL in this domain being prospectively associated with lower stress levels 1 year later. This association remained stable after controlling for gender and grade level.

**Table 6:** Longitudinal linear regression predicting perceived stress in 2021 (PSS-4, Wave 2) from baseline stress and quality-of-life (QoL) indicators (Wave 1, 2020).

Parameter	Model 1†			Model 2‡		
	Std. *β*	95% CI	*p*	Std. β	95% CI	*p*
Intercept	0.00	−0.23 – 0.23	< 0.001	0.08	−0.18 – 0.34	< 0.001
PSS-4 (Wave 1)	0.00	−0.27 – 0.28	0.970	0.01	−0.26 – 0.29	0.930
QoL autonomy and parent relation	−0.07	−0.31 – 0.16	0.550	−0.05	−0.29 – 0.18	0.650
QoL social support and peers	−0.04	−0.29 – 0.21	0.740	−0.02	−0.27 – 0.23	0.860
QoL school environment	−0.37	−0.65 – −0.09	0.010	−0.37	−0.66 – −0.08	0.010
QoL psychological well-being	−0.14	−0.41 – 0.14	0.330	−0.13	−0.42 – 0.15	0.350
Grade level	–	–	–	−0.01	−0.27 – 0.24	0.920
Gender (ref. = Male)	–	–	–			
Female	–	–	–	−0.36	−0.92 – 0.21	0.210
Observations	65			65		
*R*^2^/adj. *R*^2^	0.23/0.16			0.25/0.16		

†Model 1 includes baseline PSS-4 and all QoL predictors. ‡Model 2 additionally adjusts for grade level and gender. Std. β = standardized beta coefficient; CI = confidence interval; ref. = reference category. Observations after list-wise deletion of missing values.

### 3.2 Study 2

#### 3.2.1 Quantitative part

*RQ3*: Which school-related QoL indicators are related to stress levels in high school students after the COVID-19 pandemic?

Table 7 presents the results of a cross-sectional linear regression model examining the associations between perceived stress (PSS-4) and school-related indicators of QoL in 2023. It can be seen that low values in school-related QoL indicators (well-being, school performance, parental support, and peers) were significantly associated with higher stress levels. With the exception of school-related QoL well-being these associations remained stable after controlling for gender and grade level.

**Table 7:** Cross-sectional linear regression predicting perceived stress in 2023 (PSS-4) from school-related quality-of-life (QoL) indicators.

Parameter	Model 1†			Model 2‡		
	Std. β	95% CI	*p*	Std. β	95% CI	*p*
Intercept	−0.00	−0.12 – 0.12	< 0.001	−0.27	−0.45 – −0.08	< 0.001
School-related QoL well-being	−0.15	−0.28 – −0.03	0.020	−0.06	−0.19 – 0.08	0.420
School-related QoL school performance	−0.20	−0.33 – −0.06	< 0.001	−0.17	−0.30 – −0.03	0.014
School-related QoL parental support	−0.15	−0.27 – −0.02	0.020	−0.17	−0.29 – −0.04	< 0.001
School-related QoL peers	−0.13	−0.25 – −0.00	0.040	−0.15	−0.27 – −0.02	0.020
Grade level	–	–	–	0.05	−0.08 – 0.17	0.460
Gender (ref. = Male)	–	–	–			
Female	–	–	–	0.48	0.23–0.74	< 0.001
Diverse	–	–	–	0.02	−0.56 – 0.59	0.960
Observations	234			234		
*R*^2^/adj. *R*^2^	0.16/0.15			0.22/0.19		

†Model 1 includes only school-related QoL domains as predictors. ‡Model 2 additionally adjusts for grade level and gender (male = reference). Std. β = standardized beta coefficient; CI = confidence interval; ref. = reference category. Observations after list-wise deletion of missing values.

*RQ4*: Which school-related QoL indicators are associated with high school students’ use of school-based stress management interventions?

**Figure 2:**
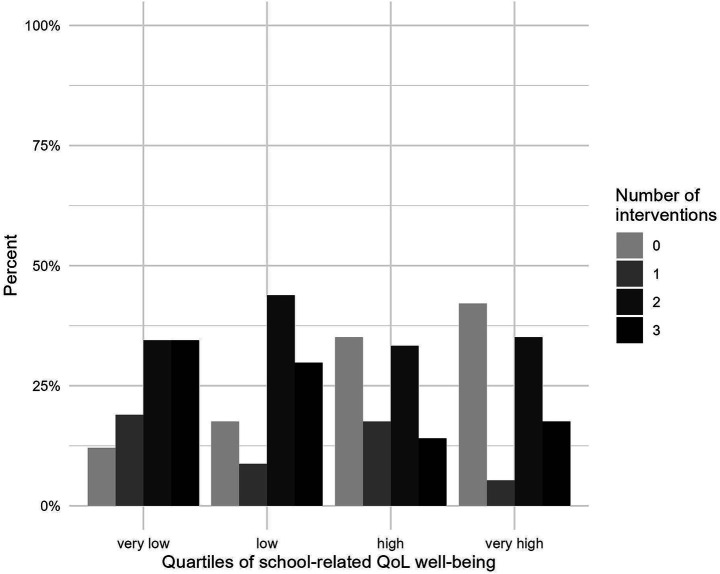
Bar chart displaying participation rates in stress management interventions at the stress management day stratified by quartiles of school-related QoL well-being. Percentages refer to the proportion of participants within each quartile of school-related QoL well-being among the cases included in the regression analyses (*N* = 229). Values 0, 1, 2, 3 represent the number of stress management activities students participated in, ranging from 0 (no intervention) to 3 (three interventions).

Table 8 presents the results of a cross-sectional linear regression model examining the associations between school-related quality of life (QoL) indicators as predictors for the use of stress management interventions. Lower school-related well-being was significantly associated with a higher number of stress management interventions partake, indicating that students with less pronounced school-related well-being tended to participate in more school-based stress management activities than students with higher well-being. This association remained stable after controlling for gender and grade level. Figure 2 shows that intervention participation varied by school-related QoL well-being. In the very low QoL quartile, 35% of students participated in three stress management activities, compared to 12% who did not participate in any activities. By contrast, in the very high QoL quartile, 42% of students did not participate in any stress management activities, compared to 18% who took part in three activities.

**Table 8:** Cross-sectional linear regression predicting students’ use of school-based stress management interventions from school-related quality-of-life (QoL) indicators (2023).

Parameter	Model 1†			Model 2‡		
	Std. β	95% CI	*p*	Std. β	95% CI	*p*
Intercept	0.00	−0.13 – 0.13	< 0.001	−0.16	−0.37 – 0.06	0.140
School-related QoL well-being	−0.21	−0.35 – −0.07	< 0.001	−0.16	−0.32 – −0.02	0.020
School-related QoL school performance	0.01	−0.13 – 0.16	0.844	0.03	−0.11 – 0.18	0.640
School-related QoL parental support	0.02	−0.11 – 0.16	0.770	0.01	−0.13 – 0.14	0.900
School-related QoL peers	−0.09	−0.23 – 0.04	0.154	−0.09	−0.23 – 0.04	0.160
Grade level	–	–	–	0.01	−0.13 – 0.15	0.890
Gender (ref. = Male)	–	–	–			
Female	–	–	–	0.27	−0.01 – 0.56	0.060
Diverse	–	–	–	0.02	−0.64 – 0.67	0.960
Observations	229			229		
*R*^2^/adj. *R*^2^	0.06/0.04			0.07/0.04		

†Model 1 includes only school-related QoL domains as predictors. ‡Model 2 additionally adjusts for grade level and gender (male = reference). Std. β = standardized beta coefficient; CI = confidence interval; ref. = reference category. Observations after list-wise deletion of missing values.

#### 3.2.2 Results – qualitative part

In the following, the results of the qualitative content analysis are presented, particularly the main categories, the related subcategories, and anchor examples for each subcategory (RQ5).

*RQ5*: Which factors facilitate the use of school-based stress management interventions?

##### 3.2.2.1 Main Category 1: Students’ overall impressions of the stress management day

The students expressed overall impressions of the stress management day that were not specific to a single session but rather reflected general aspects relevant to promoting stress management.

In general, students held a positive view of the school-based stress management day. They described the day as *relaxing*. Regardless of the specific content of individual activities, they found it refreshing to focus on stress-reduction and relaxation themes, as one student noted:

*“So I’ve also been in some courses where things were relaxed right from the start, like, basically the moment you enter the course [...] and especially the courses that were happening right in that moment, I was actually able to really switch off in those [...]. So it was a lot of fun and you could just forget everything” (K03, 03:47)*

Furthermore, students reported that the stress management day changed the perspective on experiencing stress - not only their own perspective but also the perspective, but also that of some of their teachers. On the one hand, students reported that the action day *changed their own perspective on stressful events* in the school setting. Participants described a change in cognitive appraisal such as recognizing that stress does not need to be experienced as an uncontrollable condition but can, in fact, be actively managed.

*“So in general, it was also a lot about a positive attitude. [...] You can't change things like that. For example, you have to go to school, and then it depends a lot on the attitude. If you say, ‘Yeah, cool, I'm going to school now,’ and you kind of see the positive things, then it's much easier than if you say, ‘How stupid is this now?’ or something like that.” (K06, 18:47)*

On the other hand, students reported that the day also seemed to have *changed perspective of teachers on students’ stress experiences* in the school setting, as they showed increased interest in students’ everyday stressors in school.

*“Several teachers, whom we had in regular classes that day, also asked what is actually so stressful in our everyday life [.]. And I think it’s actually quite good that the teachers know, because maybe they can help us a little and support us more, rather than not knowing why we are stressed at all.” (K02, 33:58)*

The students emphasized that having the opportunity to engage in *stress management activities within the school setting, to which they had had little exposure up to now*, was beneficial. The students emphasized that it was beneficial to have been introduced to topics such as relaxation techniques within the school setting. Several respondents stated that, until now, they had had little exposure to these subjects in their everyday lives. As such, they appreciated the school’s role in presenting these topics, particularly given the importance of not only meeting academic demands but also maintaining mental health. One student summed it up:

*“I believe very few students would engage with this voluntarily in their private lives or actively sign up for something like this on their own. It’s simply more accessible when it’s offered in school, as it provides an opportunity to explore it in a familiar setting”* (K05, 7:03)

Students stated that such stress management days are a significant *change from the regular school routine*. They found it enriching to engage in activities that differed notably from their usual school day. One student stated:

*“It wasn’t explicitly about learning this time, but about trying something new." (K05, 5:55)*

##### 3.2.2.2 Main category 2: Factors that facilitate the implementation of stress management methods in students’ daily lives

Some students reported that they had already applied methods they had learned on the stress management day. In their reflections, they explained what had facilitated this transfer. The subcategories presented below are based on these explanations and refer to the transfer of specific new methods learned during the activities.

Students emphasized that *having fun* during the intervention was an important facilitating factor. For example, the course instructors led the yoga exercises in an engaging and enjoyable way, which motivated students to try the exercises at home—sometimes even practicing together with their siblings.

*"I became so bored while doing my homework that I could no longer concentrate properly. So I thought to myself, 'Why not do something fun instead?' Then I remembered the exercises we had done during the yoga session. I ended up doing those exercises either alone or together with my sister. […] One of us had to get into a position on hands and feet, while the other stood on their back with their feet, placing their hands on the ground in front. It looked quite funny."* (K04, 21:47)

Students reported that *Easy and Quick to Learn Methods* for stress management that can be learned within the short time frame of an action day are particularly useful. This was generally the case with breathing and relaxation exercises offered in various programs, such as Progressive Muscle Relaxation (PMR), as described by one student:

*“The relaxation techniques we practiced during the training [...] because they are quick to do, but they were definitely effective.” (K05, 15:42)*

The students reported that breathing exercises, as well as the muscle exercises of PMR, are easily *transferable into everyday life* and are therefore well-suited for school-based action days on stress management. Learning the breathing exercises takes only a few minutes, and the relaxation effect is immediately noticeable, as one student describes:

*“Because it's much easier and also very short. I mean, it's really not a big effort to do it. […] It only takes less than a minute”.*

Students indicated that *clear and understandable objectives* of a stress management activity right from the outset of the intervention are essential. This was, for example, achieved by allowing participants to directly experience how the activity contributed to stress reduction. Additionally, it should become evident right at the beginning how the method can be applied in everyday life. This, for example, is the case with PMR, as one student explains:

*“You really get the impression several times, especially during the introduction, of how it works, and [...] if you don't immediately absorb it at the beginning, then you don't know what the feeling is like, and if you have to learn it later on, you just won't do it.” (K03, 20:18)*

## 4 Discussion

### 4.1 Principal findings

This study examined patterns of perceived stress among German high-school students during and after the COVID-19 pandemic (2020–2021). We observed significant fluctuations in stress levels over this period. These findings align with earlier research reporting associations between pandemic related disruptions such as social isolation and interrupted schooling and elevated stress and mental health concerns among students (6).

Second, we investigated whether interindividual differences in quality of life (QoL) indicators across various life domains were associated with perceived stress. Across both cross-sectional and longitudinal analyses, higher school-related QoL was consistently linked to lower perceived stress, suggesting a potentially protective association that extends over time. The findings suggest that higher school-related QoL may be associated with lower stress levels and could represent a potential protective factor of lower stress during the pandemic, echoing previous reports (9, 10). Building on the transactional model of stress (2), these results may indicate that students with better school-related QoL tend to appraise potentially stressful situations as less relevant to their personal well-being or more as a positive challenge during primary appraisal, whereas those with lower school-related QoL may perceive such potential stressors as more threatening to their well-being, which is reflected in higher reporte stress. Furthermore, students reporting higher school-related QoL may also perceive more resources at school, such as increased social support from friends or teachers or higher self-efficacy due to good grades and thus assess their ability to successfully manage stressful situations during secondary appraisal as higher than students with lower school-related QoL who may have less confidence in their ability to deal with stressful situations at school, due to a lack of perceived resources. This may have been particularly relevant after the onset of the pandemic, with home-schooling being implemented and the consequent loss of a potent coping strategy, namely social support. Although many social interactions moved to online platforms, online social support has been found to be less effective for adolescents than social support received in person (37).

Third, our mixed-methods research aimed to identify associations of school-related indicators of QoL with perceived stress on the one hand and with the use of school-based stress management interventions on the other hand. Lower school-related well-being appeared to be linked with a greater likelihood of engaging in stress management interventions. It is possible that the observed association may reflect a tendency for students with lower school-related QoL to participate voluntarily in stress management interventions, potentially perceiving such activities as opportunities to enhance their school-related well-being. In line with the transactional model of stress and coping (2), students with lower school-related QoL may appraise their ability to cope stressful situations as lower, and may therefore perceive greater benefit from stress management interventions than students with higher school-related QoL. This aligns with our qualitative data, showing that stress management days at school have the potential to offer students’ insights into different stress management strategies. Focus group insights highlighted the perceived value of stress management days, suggesting that experiential, hands-on approaches can enhance students’ engagement with coping strategies. Furthermore, the results indicated that the stress management day contributed to a shift in perspectives on stress within the school setting among students and teachers. Building on the transactional model of stress (2), these positive experiences made during the school-based stress management interventions may expand students’ perceived resources. This may be associated with more adaptive coping strategies and may potentially impact the appraisal of school-based stressors as more manageable, thus reducing perceived stress. Finally, the focus groups indicated that the implementation of stress management strategies into schools’ daily routines may be facilitated by factors such as enjoyment, the availability of methods that are quick to learn and easy to implement, and activities with clear and understandable goals (20, 21). Thus, linking school-related indicators for QoL with stress levels in high school students and with the use of stress management interventions might be a fruitful avenue for future stress research (38, 39). Taken together, the quantitative and qualitative findings from Study 2 offer complementary insights into students’ engagement with school-based stress management interventions. The quantitative results indicated that lower school-related QoL was associated with greater participation, while the qualitative findings highlighted contextual factors - such as enjoyment, ease of implementation in everyday life - that facilitate students’ use of these interventions. Considering both strands jointly suggests that effective stress management initiatives at school should not only target students who report lower school-related well-being but also emphasize engaging, accessible and practically oriented components that encourage continued participation.

In line with previous research on gender-specific coping responses both in adults (3) and school-aged children (40), we included gender as a covariate in our statistical models. Future school-based stress management programs may address gender-related variations in coping, promote mutual learning among students, and broaden their repertoire of effective stress management strategies. Overall, the results emphasize the important role of schools as key settings for supporting students in coping with stress. This can be achieved by organizing dedicated stress management days that differ from regular school routines and provide opportunities to explore various easy-to-learn coping methods. Beyond individual schools, these findings underscore the need for sustained efforts in research and policy to strengthen students’ stress management competencies. One initiative moving in this direction is the IMPROVA (2025) project, which aims to improve adolescent mental health and well-being through a co-created eHealth intervention. Funded by Horizon Europe, *IMPROVA* focuses on early detection, stigma reduction, and mental health promotion in secondary schools across four European countries. The project uses a stepped-wedge randomized design to evaluate an intervention platform tailored to students, teachers, parents, and school health professionals. By incorporating socio-cultural factors and previous best practices, *IMPROVA* seeks to identify high-risk groups and deliver scalable, context-sensitive support. This broader European initiative aligns with our findings, highlighting the relevance of school-related QoL and demand-tailored stress interventions in supporting adolescents’ mental health.

### 4.2 Limitations

#### 4.2.1 Quantitative data

There are various limitations of this study. Due to the research design, it cannot be inferred that it was specifically school-related QoL indicators that increased the motivation to partake in a stress management intervention. There might be several confounders that influenced this result (e.g, socio-economic status, cognitive abilities). A major limitation of this study is the low internal validity resulting from the absence of a control condition and the lack of pre-pandemic baseline data collected at the same school. Without these elements, causal inferences regarding the impact of the COVID-19 pandemic on students’ stress levels and quality of life as well as the specific effects of the stress management day remain limited. Future research should therefore employ more rigorous designs to strengthen causal interpretation, including a broader sampling frame with more schools. In particular, randomized controlled trials (RCTs) or quasi-experimental studies including a control group, for example, a comparable school that does not implement a stress management intervention day would allow for more robust conclusions about intervention effects.

Another limiting factor is that the sample was not recruited randomly, but rather at one high school. This narrow sampling frame and the small longitudinal sample size limit the representativeness of the findings and raise questions about generalizability. In particular, potential biases related to the socio-economic background, academic track of the school or pre-existing mental health conditions cannot be excluded. Since data collection was conducted under conditions of strict anonymity, it is not possible to further characterize the sample using socioeconomic or other background indicators. Consequently, the findings should be interpreted with caution and seen as preliminary, requiring replication in more diverse samples across different school types and contexts.

The psychometric properties of the PSS-4 have been questioned (31). In our sample, corrected item–total correlations exceeded conventional thresholds (41); however, internal consistency estimates were modest for PSS-4 (Cronbach’s *α* and McDonald’s *ω*), which may have attenuated effect estimates. This pattern is common for very brief psychometric instruments, for which Cronbach’s α and McDonalds ω often underestimate reliability (4, 42). Consistent with prior work, other studies using the PSS-4 have reported similarly modest α values (43). Although consistent with prior work some of the KIDSCREEN short form subscales had modest internal consistency estimates (44). These psychometric constraints should be considered when interpreting the results.

Furthermore, results would be more robust when a self-report like the PSS-4 is combined with other stress assessment methods such as the Mobile Photographic Stress Meter (45) and objective measures, sensors, and computational methods (46) such as information gained from ecological monitoring assessment (47, 48) and crowd sensing passive mobile phone sensing (49). Integrating such different measures of stress should be a next step in research on changes in stress levels in high school students.

#### 4.2.2 Qualitative data

Because our sample came from a single school, the findings cannot be assumed to generalize to other settings. Nevertheless, students’ overall impressions of the stress management day as well as factors that facilitate the implementation of stress management methods in students’ daily lives in the focus groups were not school-specific and are therefore likely transferable to a broader high school student population. With six focus groups with up to 12 students across study phases, the qualitative sample was still relatively small and non-representative. Even so, thematic saturation was achieved. Small focus-group sizes, while a limitation, also fostered a supportive atmosphere that prompted most students to speak.

We chose qualitative content analysis for its theory-guided, systematic handling of large textual material (34). However, the method’s category-based reduction can result in a loss of nuance, potentially diminishing the richness of individual cases and perspectives (34). Additionally, qualitative content analysis has limitations in exploring deeper, latent structures within texts (34). However, such in-depth interpretation was not the aim of this study. Another potential limitation is that, although two coders were involved in the inductive coding process, the selection of text segments was carried out by only one coder.

As the current study did not integrate qualitative and quantitative data to address the same research question, future research should link the qualitative and quantitative data of individual participants using personalized codes as unique identifiers.

## 5 Conclusion

This study demonstrated that perceived stress levels among German high-school students increased during and after the COVID-19 pandemic and that this increase was associated with lower quality of life (QoL) across several life domains. Moreover, low school-related well-being was associated with the use of school-based stress management interventions. These findings suggest that high schools can help students cope with school-related stress by planning and implementing stress management action days.

## Data Availability

The raw data supporting the conclusions of this article will be made available by the authors, without undue reservation.
