# Supplementary material for: Quality of life, perceived stress, and use of school-based stress management interventions in high school students: a mixed-methods study during and after COVID-19
Source: Front Public Health. 2025 Dec 11;13:1658346. doi: 10.3389/fpubh.2025.1658346 (PMC12738349; doi:10.3389/fpubh.2025.1658346)
Supplement: Supplementary file 1 [file Supplementary_file_1.docx]

**Appendix 1.** Reliability coefficients for the PSS-4 scale.

| **Metric** | **Wave 1**  **(2020, study 1)** | **Wave 2**  **(2021, study 1)** | **Stress Management Day**  **(study 2)** |
| --- | --- | --- | --- |
| Cronbach’s α | 0.60 | 0.75 | 0.61 |
| McDonald’s ω | 0.61 | 0.76 | 0.61 |
| Item–total correlation (min–max) | 0.34–0.41 | 0.48–0.62 | 0.34–0.45 |

*Notes.* PSS-4 = 4-item Perceived Stress Scale.
