# Supplementary material for: Quality of life, perceived stress, and use of school-based stress management interventions in high school students: a mixed-methods study during and after COVID-19
Source: Front Public Health. 2025 Dec 11;13:1658346. doi: 10.3389/fpubh.2025.1658346 (PMC12738349; doi:10.3389/fpubh.2025.1658346)
Supplement: Supplementary file 2 [file Supplementary_file_2.docx]

**Appendix 2.**

**Variance Inflation factors (VIF), residuals-versus-fitted plots and Q-Q plots for the cross-sectional linear regression predicting perceived stress (PSS-4) from Quality of Life (QoL) indicators, Wave 1 (2020)**

| Variable | VIF (Model 1)† | VIF (Model 2)‡ |
| --- | --- | --- |
| QoL Autonomy & Parent Relation | 1.17 | 1.17 |
| QoL Social Support & Peers | 1.20 | 1.21 |
| QoL School Environment | 1.38 | 1.51 |
| QoL Psychological Well-being | 1.32 | 1.34 |
| Grade level | — | 1.06 |
| Gender | — | 1.09 |

**Model 1†**

**
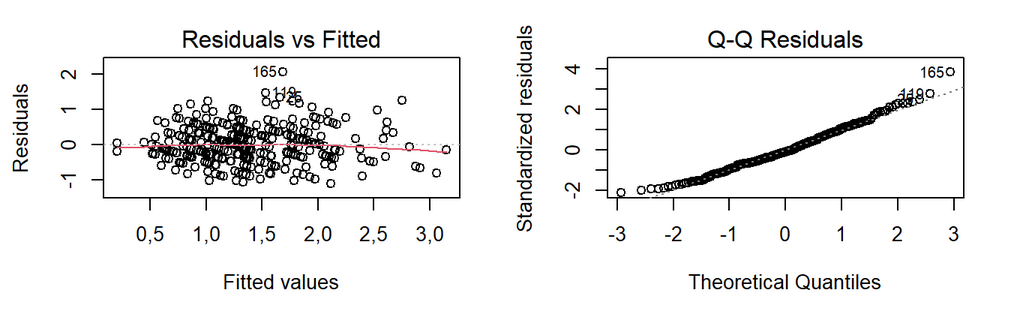
**

**Model 2‡**

**
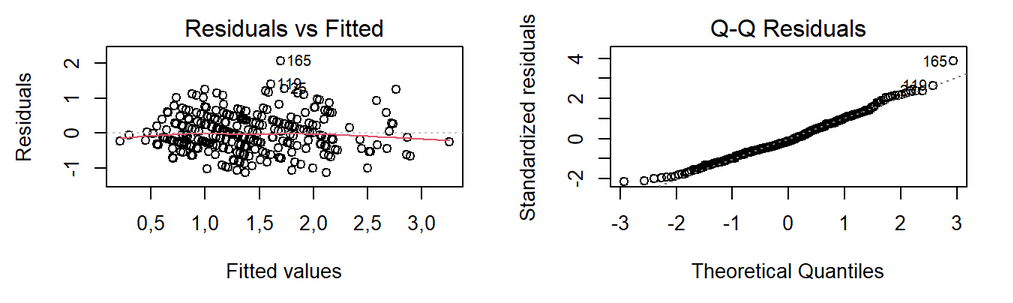
**

**Variance Inflation factors (VIF), residuals-versus-fitted plots and Q-Q plots for the longitudinal linear regression predicting perceived stress in 2021 (PSS-4, Wave 2) from baseline stress and quality-of-life (QoL) indicators (Wave 1, 2020)**

| Variable | VIF (Model 1)† | VIF (Model 2)‡ |
| --- | --- | --- |
| PSS-4 (Wave 1) | 1.44 | 1.45 |
| QoL Autonomy & Parent Relation | 1.06 | 1.07 |
| QoL Social Support & Peers | 1.19 | 1.21 |
| QoL School Environment | 1.48 | 1.60 |
| QoL Psychological Well-being | 1.47 | 1.51 |
| Grade level | — | 1.22 |
| Gender | — | 1.04 |

**Model 1†**

**
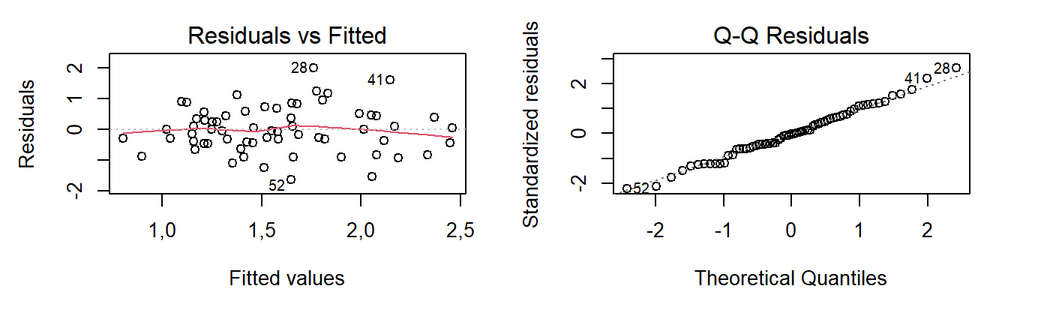
**

**Model 2‡**

**
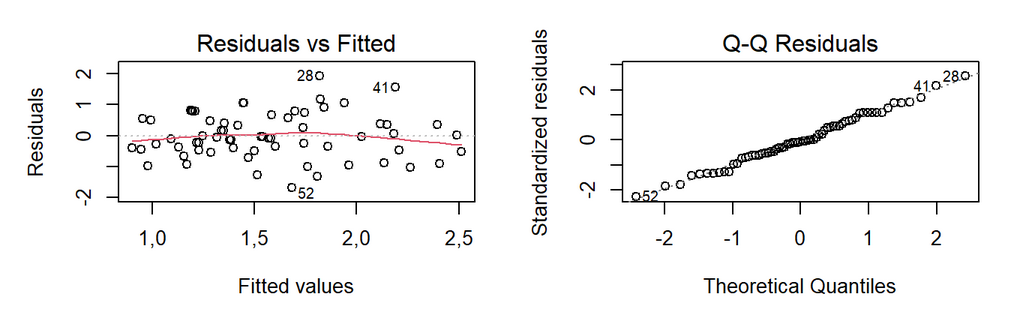
**

**Variance Inflation factors (VIF), residuals-versus-fitted plots and Q-Q plots for the cross-sectional linear regression predicting perceived stress in 2023 (PSS-4) from school-related quality-of-life (QoL) indicators**

| Variable | VIF (Model 1)† | VIF (Model 2)‡ |
| --- | --- | --- |
| School-related QoL well-being | 1.12 | 1.38 |
| School-related QoL school performance | 1.26 | 1.29 |
| School-related QoL parental support | 1.12 | 1.14 |
| School-related QoL peers | 1.08 | 1.10 |
| Grade level | — | 1.13 |
| Gender | — | 1.20 |

**Model 1†**

**
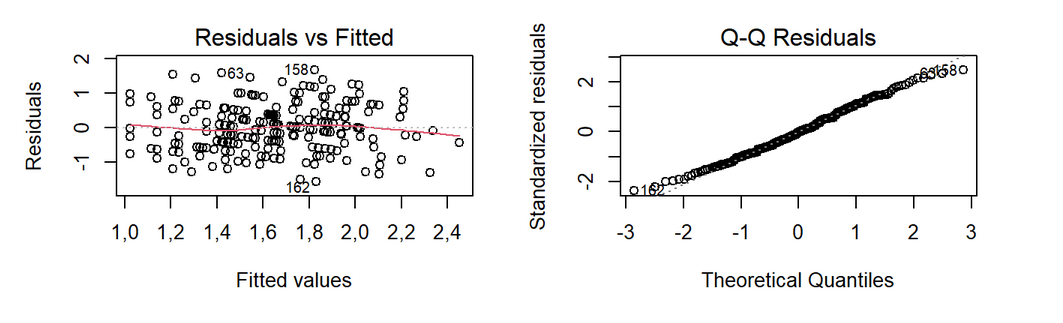
**

**Model 2‡**

**
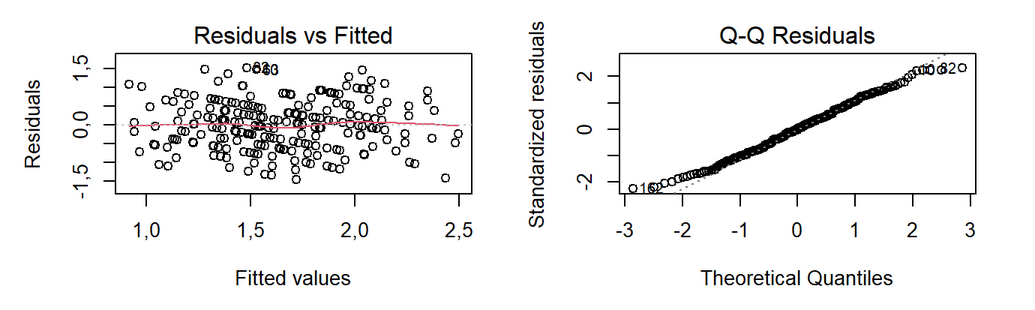
**

**Variance Inflation factors (VIF), residuals-versus-fitted plots and Q-Q plots for the cross-sectional linear regression predicting students’ use of school-based stress management interventions from school-related quality-of-life (QoL) indicators (2023)**

| Variable | VIF (Model 1)† | VIF (Model 2)‡ |
| --- | --- | --- |
| School-related QoL well-being | 1.14 | 1.35 |
| School-related QoL school performance | 1.25 | 1.31 |
| School-related QoL parental support | 1.12 | 1.14 |
| School-related QoL peers | 1.04 | 1.07 |
| Grade level | — | 1.14 |
| Gender | — | 1.18 |

**Model 1†**

**
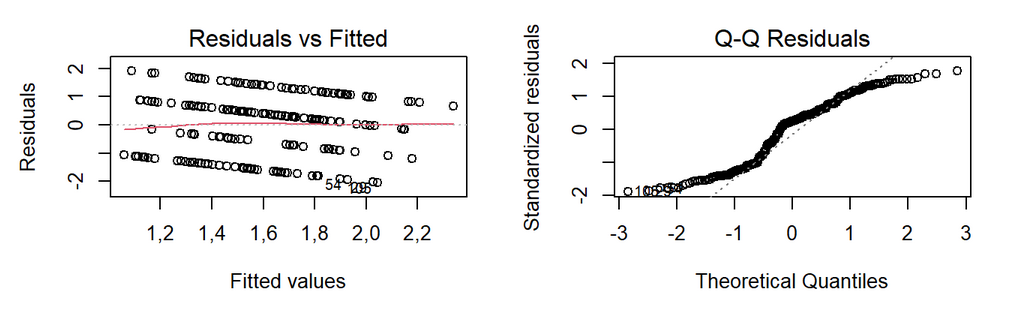
**

**Model 2‡**

**
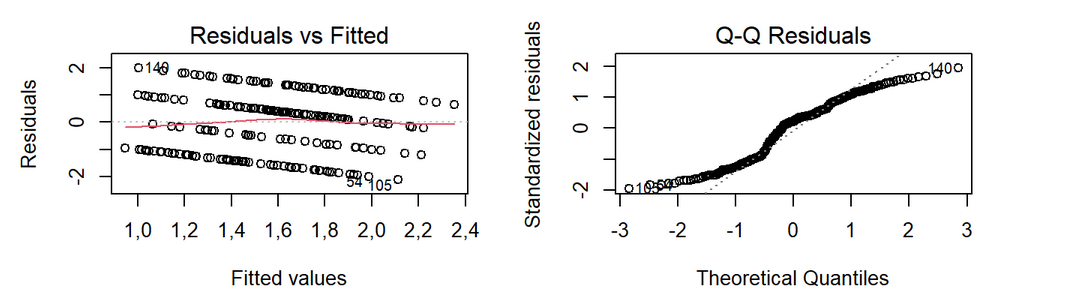
**
