# Supplementary material for: Quality of life, perceived stress, and use of school-based stress management interventions in high school students: a mixed-methods study during and after COVID-19
Source: Front Public Health. 2025 Dec 11;13:1658346. doi: 10.3389/fpubh.2025.1658346 (PMC12738349; doi:10.3389/fpubh.2025.1658346)
Supplement: Supplementary file 3 [file Supplementary_file_3.docx]

**Appendix 3. Per-variable missingness by study**

| **Variable** | **Wave 1 (2020) N=345** | **Wave 2 (2021) N=217** | **Stress Management Day (2023) N=281** |
| --- | --- | --- | --- |
| PSS-4 | 17 (4.93%) | 9 (4.15%) | 22 (7.83%) |
| School-related QoL: well-being | — | — | 12 (4.27%) |
| School-related QoL: school performance | — | — | 2 (0.71%) |
| School-related QoL: parental support | — | — | 6 (2.14%) |
| School-related QoL: peers | — | — | 4 (1.42%) |
| QoL: Autonomy & Parent Relation | 5 (1.45%) | — | — |
| QoL: Social Support & Peers | 16 (4.64%) | — | — |
| QoL: School Environment | 7 (2.03%) | 3 (1.38%) | — |
| QoL: Psychological Well-being | 12 (3.48%) | 12 (5.53%) | — |
| Use of school-based stress management interventions | — | — | 25 (8.90%) |
| Grade level | 10 (2.90%) | — | 6 (2.14%) |
| Gender | 0 (0.00%) | — | 4 (1.42%) |

**Notes.** Missing values are n (%). Per-variable missingness percentages are calculated using the wave-specific total N shown in the header row. “—” indicates the variable was not collected.

Little’s test for Missing Completely at Random (MCAR) was non-significant in all datasets: Wave 1, χ²(54) = 68.6, p = .09, Wave 2 χ²(7) = 10.6, p = .16 and the Stress Management Day assessment χ²(101) = 117.0, p = .13. These results support the MCAR assumption; accordingly, we used listwise deletion in the regression analyses.
