# Supplementary material for: Quality of life, perceived stress, and use of school-based stress management interventions in high school students: a mixed-methods study during and after COVID-19
Source: Front Public Health. 2025 Dec 11;13:1658346. doi: 10.3389/fpubh.2025.1658346 (PMC12738349; doi:10.3389/fpubh.2025.1658346)
Supplement: Supplementary file 4 [file Supplementary_file_4.docx]

Appendix 4: stress management activities offered at the stress management day

| **stress management activities** | **Duration for each session** | **Number of sessions per day** | **Maximum of participants per session** | **Maximum number of participants** | **Participant attendance rates*** |
| --- | --- | --- | --- | --- | --- |
| AcroYoga | 60 min. | 2 | 10 | 20 | 26 |
| Aqua Gym | 60 min. | 2 | 20 | 40 | 38 |
| Line Dance | 60 min. | 3 | 50 | 150 | 21 |
| Mindfulness and awareness | 60 min. | 3 | 10 | 30 | 30 |
| Progressive Muscle Relaxation | 60 min. | 3 | 25 | 75 | 55 |
| Qigong | 45 min. | 3 | 15 | 45 | 38 |
| Saluté – Stress Management Program | 60 min. | 3 | 15 | 45 | 19 |
| Strategies for coping with exam anxiety | 60 min. | 3 | 10 | 30 | 28 |
| TrophoTraining | 60 min. | 3 | 20 | 60 | 56 |
| Yoga – Course 1 | 60 min. | 3 | 15 | 45 | 35 |
| Yoga – Course 2 | 60 min. | 3 | 12 | 36 | 32 |
| Zumba | 30 min. | 2 | 15 | 30 | 18 |

* Attendance rates are based only on students who answered the questionnaire (see Methods section, study 2); actual participation may be higher (We have no information about those students who did not answer the questionnaire)

The stress management activities were conducted in separate rooms within the school building. All activities took place concurrently on the stress management day. Most activities were offered in three sessions, starting at 8:45 a.m., 10:00 a.m., and 11:15 a.m., allowing students to participate in up to three sessions. Each time slot was followed by a 15- to 45-minute break, depending on the duration of the preceding activity. Participation was voluntary, and students were permitted to decline participation in activities. In this case, they were required to attend classes regularly.
